# Tolerability of eptinezumab in overweight, obese or type 1 diabetes patients

**DOI:** 10.1002/edm2.217

**Published:** 2021-02-02

**Authors:** Brian Baker, Barbara Schaeffler, Joe Hirman, Marcus Hompesch, Susan Pederson, Jeff Smith

**Affiliations:** ^1^ Lundbeck Seattle BioPharmaceuticals, Inc. Bothell WA USA; ^2^ Pacific Northwest Statistical Consulting Woodinville WA USA; ^3^ ProSciento, Inc. Chula Vista CA USA; ^4^ Alder BioPharmaceuticals, Inc. (now known as Lundbeck Seattle BioPharmaceuticals, Inc.) Bothell WA USA

**Keywords:** eptinezumab, obesity, type 1 diabetes

## Abstract

**Introduction:**

In addition to its role in the pathogenesis of migraine, calcitonin gene‐related peptide (CGRP) is implicated in the regulation of insulin secretion. However, there are limited data on the use of CGRP inhibitor monoclonal antibodies in individuals who are overweight/obese and those with diabetes.

**Methods:**

Two randomized, double‐blind, placebo‐controlled trials were conducted to assess the safety and metabolic effects of eptinezumab in non‐migraine overweight/obese patients (study 1) and patients with type 1 diabetes (T1D; study 2). The primary end‐point in overweight/obese patients was safety and changes in basal metabolic rate (BMR), defined as the energy expenditure during the fasting and resting states. In patients with T1D, the primary end‐points were safety and insulin sensitivity as assessed by the bodyweight and insulin concentration corrected glucose infusion rate (M/I).

**Results:**

A total of 24 patients were enrolled in study 1, and 21 patients were enrolled in study 2. In overweight/obese patients, there was no significant difference in the least squares (LS) mean change in BMR between the eptinezumab‐ and placebo‐treated patients from baseline to day 7 (6.4 vs −25.2 Kcal/day; LS mean difference 31.6 [95% confidence interval −90.6, 153.8]). In patients with T1D, there was no significant difference in insulin sensitivity between the eptinezumab and placebo groups. Eptinezumab was well tolerated in both studies with a similar rate of adverse events between treatment groups, and no new safety signals were identified.

**Conclusion:**

Eptinezumab was well tolerated and not associated with adverse metabolic effects in patients who were overweight/obese or had T1D, providing ongoing support for the use of eptinezumab in these subgroups of patients with migraine.

## Graphical abstract

Two phase 1 studies evaluated the safety and metabolic effects of eptinezumab (a calcitonin gene‐related peptide [CGRP] inhibitor) in non‐migraine overweight/obese patients (study 1) and non‐migraine patients with type 1 diabetes (T1D; study 2), given that CGRP has also been implicated in the regulation of insulin secretion. In both studies, eptinezumab was well tolerated and not associated with adverse metabolic effects, providing support for the use of eptinezumab in these subgroups of patients with migraine.

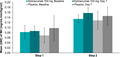

## 1 INTRODUCTION

Migraine is a neurological disorder and a leading cause of long‐term disability, especially in women.^1^, ^2^ In recent years, the calcitonin gene‐related peptide (CGRP) has been implicated in the pathophysiology of migraine^3^, ^4^; serum concentrations of CGRP have been found to be elevated during migraine headache,^5^, ^6^ leading to sensitization of the trigeminal system.^7^ These observations sparked the development of small molecule antagonists and monoclonal antibodies (mAbs) directed towards inhibiting CGRP activity,^3^, ^4^ with several agents now in late stage clinical development or recently approved for treatment.^8^ In addition to its role in migraine, animal models indicate a link between CGRP and the regulation of insulin and glucagon secretion.^9^, ^10^, ^11^ However, the relationship between migraine in obese patients and glucose metabolism is complex, with data that are conflicting. Insulin sensitivity has been shown to be impaired in patients with migraine,^12^ and additionally, abdominal obesity is a significant risk factor for insulin resistance in women with and without migraine.^13^ Although most evidence suggests that obesity is not associated with an increased prevalence of migraine, obesity does appear to be associated with increased migraine frequency (eg in the transformation from episodic to chronic migraine).^14^, ^15^, ^16^ An evaluation of 30 215 subjects with a history of headache, including 3719 with migraine, found no effect of body mass index (BMI) on migraine prevalence; nonetheless, a progressive increase in the proportion of subjects who experienced 10–14 headache days per month between normal weight, overweight, obese and severely obese groups was observed.^15^ Furthermore, weight loss has been shown to be associated with a reduction in headache frequency, severity and disability.^17^

The mechanisms underlying the relationship between migraine and obesity are not completely understood, but multiple factors appear to be involved, including elevated levels of pro‐inflammatory mediators (eg c‐reactive protein, tumour necrosis factor, interleukin‐6, mast cells) as well as elevations in CGRP and related neuropeptides (ie amylin, adrenomedullin).^15^ Notably, CGRP also appears to play a role in the pathogenesis of diabetes; CGRP levels are elevated in obese and diabetic rat models, and the administration of capsaicin, which mediates CGRP‐expressing sensory fibres in islet cells, lowers blood glucose after an oral glucose tolerance test and improves insulin sensitivity.^10^, ^11^ In addition, when fed a high‐fat diet, mice lacking α‐CGRP have improved insulin sensitivity and glucose handling compared with wild‐type mice, including protection against diet‐induced obesity.^9^ In support of this hypothesis, clinical data have also indicated a link between migraine and diabetes, with one large study reporting a 40% increased risk of migraine among those with diabetes.^18^

Eptinezumab is a humanized mAb that inhibits CGRP; it selectively binds to CGRP with high affinity, resulting in rapid inactivation of CGRP activity and signalling.^19^ In the phase 3 PROMISE‐1 and PROMISE‐2 studies, eptinezumab demonstrated a statistically significant reduction in mean migraine days versus placebo.^20^, ^21^ Eptinezumab was also well tolerated in these studies, with an overall incidence of adverse events (AEs) that was similar to that of placebo‐treated patients.

Currently, there are limited data on the use of CGRP inhibitor mAbs in overweight or obese persons, patients with diabetes or subpopulations with metabolic abnormalities that include impaired glucose handling and insulin insensitivity. Here, therefore, we present two randomized, double‐blind, placebo‐controlled, parallel‐group studies designed to assess the safety and metabolic effects of eptinezumab treatment in non‐migraine overweight/obese patients (study 1) and to assess safety and changes in insulin sensitivity and daily insulin requirements in patients with type 1 diabetes (T1D; study 2).

## 2 MATERIALS AND METHODS

### 2.1 Non‐migraine overweight or obese patients (study 1)

#### 2.1.1 Patients

The study included patients between the ages of 18 and 45 years, who were overweight or obese but otherwise healthy. Inclusion criteria required patients to have a stable BMI, during the 2 months prior to screening, of between ≥25.0 and <40.0 kg/m^2^. Exclusion criteria included a history of diabetes, or current evidence of any clinically significant medical condition, psychiatric condition, or observed abnormality that could potentially compromise patient safety or affect the pharmacodynamic evaluations. Chronic use of nonsteroidal anti‐inflammatory drugs (NSAIDs) and acetaminophen was not permitted, with the exception of low‐dose aspirin. The use of all other prescription medication, over‐the‐counter medications (including opioids), and natural health products was prohibited, with the exception of oral contraceptives.

#### 2.1.2 Study design

This was a phase 1, double‐blind study. After screening, patients were randomly assigned in a 2:1 ratio to receive either eptinezumab 100 mg or placebo. Treatment was administered intravenously (IV) over a 1‐h period. Three follow‐up visits were planned, at 4, 8 and 12 weeks.

Primary end‐points were to evaluate the safety of eptinezumab and to assess any changes in basal metabolic rate (BMR). AEs were recorded throughout the course of the study and were assessed for severity and causality, with a focus on treatment‐emergent adverse events (TEAEs). Other safety end‐points included the occurrence of clinical laboratory abnormalities, change from baseline in vital signs and in 12‐lead electrocardiogram (ECG) parameters, presence of suicidal ideation and development of anti‐drug and neutralizing antibodies. BMR was determined by indirect calorimetry and was defined as the energy expenditure measured during fasting (ie 0‒30 min preprandial) and resting states.

Secondary pharmacodynamic end‐points assessed changes in substrate oxidation (respiratory quotient, diet‐induced thermogenesis and urinary nitrogen excretion), ad libitum food intake/feelings of hunger and satiety, and postprandial metabolism of glucose and lipid.

### 2.2 Patients with T1D (study 2)

#### 2.2.1 Patients

This study included male or female patients, aged 18–45 years, with an established diagnosis of T1D, and who were receiving stable insulin therapy (<1.2 IU/kg of total insulin per day) via a continuous subcutaneous insulin infusion (CSII) pump. Patients were required to have a BMI between 18 and 30 kg/m^2^ inclusive, and they must have had a stable weight with no more than 5% gain or loss in the 3 months prior to screening. Patients were also required to have been prescribed insulin for at least 1 year before screening, to have received insulin treatment within 6 months of the T1D diagnosis and to have glycosylated haemoglobin (HbA1c) ≤9.5% at screening. Exclusion criteria included a history, within 6 months prior to entry into the study, of either ≥2 episodes of severe hypoglycaemia, or ≥1 episode of diabetic ketoacidosis, or ≥1 emergency room visit for uncontrolled diabetes which resulted in hospitalization. Prohibited medications included anti‐hyperglycaemic medications other than insulin, drugs that could influence glucose metabolism (eg systemic corticosteroids, monoamine oxidase inhibitors, prostaglandin blockers, systemic non‐selective beta‐blockers, growth hormone) and weight‐lowering medications.

#### 2.2.2 Study design

This was a randomized, double‐blind, placebo‐controlled, parallel‐group study. After screening, patients were randomly assigned in a 2:1 ratio to receive either eptinezumab 100 mg or placebo; both were administered IV over a 1‐h period. Four follow‐up visits were conducted, at 4, 8, 12 and 24 weeks.

The primary end‐points for this study were safety and an assessment of insulin sensitivity. Safety end‐points included the incidence, severity and causality of TEAEs, occurrence of clinical laboratory abnormalities and changes from baseline in vital signs and 12‐lead ECG. To measure whole‐body insulin sensitivity, patients underwent a two‐step hyperinsulinemic euglycemic glucose clamp procedure pre‐ and post‐treatment. At least 2 h prior to the start of the clamp procedure, patients were connected to an automated glucose analyser, which calculated the glucose infusion rate (GIR) required to maintain the target blood glucose level at 100 mg/dl. During the clamp procedure, insulin lispro was infused in two consecutive steps, with each step lasting approximately 180 min. In Step 1, insulin was infused at a rate of 10 mU/m^2^/min for 180 min; this raised the plasma insulin concentration to a level suitable for the assessment of endogenous glucose production (EGP) sub‐maximal suppression. Immediately following Step 1, Step 2 was initiated, during which insulin was infused at a rate of 40 mU/m^2^/min for 180 min. This level of insulin infusion was sufficient to provide near‐maximal glucose disposal and EGP suppression. Blood samples to measure plasma glucose levels were drawn before and during the clamp procedure. The primary metabolic end‐point was M/I, defined as the change from baseline to day 7 in the weight‐corrected GIR divided by the plasma insulin concentration at steady state. The assessment of insulin sensitivity also included the change from baseline to day 7 in the bodyweight‐corrected GIR and changes in the insulin sensitivity index (SI). The insulin SI was defined as the mean change in GIR between Step 1 and Step 2 of the clamp procedure divided by the difference in steady‐state insulin concentrations between Steps 1 and 2, corrected for the average steady‐state blood glucose concentration. Total daily insulin requirements were also assessed.

### 2.3 Ethical considerations

Both studies were performed in accordance with the ethical principles stated in the latest version of the Declaration of Helsinki and the applicable guidelines of Good Clinical Practice and were approved by the investigational review board. All patients, in both studies, provided written informed consent.

### 2.4 Statistical analysis

Adverse events were summarized descriptively in both studies. For categorical variables, the frequency and percentage in each category were to be provided. In study 1, change from baseline to day 7 in BMR was evaluated using an analysis of covariance (ANCOVA) model, with treatment as a factor and baseline BMR value as a covariate. The least squares (LS) mean, standard error (*SE*) and 95% confidence interval (CI) were calculated for each treatment group; the LS mean difference between eptinezumab‐ and placebo‐treated patients, the 95% CI and the *SE* of the difference were also calculated. In study 2, the change in M/I from baseline to day 7 was analysed using an ANCOVA model, with treatment as a factor and baseline value of M/I as a covariate. The LS mean, *SE* and 95% CI were calculated for each treatment group and clamp procedure period. In addition, the LS mean difference between eptinezumab and placebo, the associated 95% CI and the *SE* of the difference were calculated. Testing for significance was not conducted in either study.

## 3 RESULTS

### 3.1 Healthy overweight or obese patients (study 1)

#### 3.1.1 Patient demographics and disposition

A total of 24 patients (16 eptinezumab, 8 placebo) were enrolled into study 1, and demographic characteristics are shown in Table 1. The mean age was 35.3 years in the eptinezumab group and 32.9 years in the placebo group; the majority of patients in both groups (62.5%) were male. Four patients (two from each treatment group) withdrew from the study, after receiving treatment (between days 10 to 57) but prior to completion of the study. All four patients withdrew for personal reasons related to moving out of the local area, and all were included in both the safety and pharmacodynamic evaluations. Seven of 24 patients were taking concomitant medications, the most common of which was ibuprofen (*n* = 3). No other concomitant medication was taken by more than one patient.

**TABLE 1:** Patient demographics

Parameter	Study 1 (Overweight/Obese)		Study 2 (T1D)	
	Eptinezumab 100 mg *N* = 16	Placebo *N* = 8	Eptinezumab 100 mg *N* = 14	Placebo *N* = 7
Age (years)	35.3 ± 6.0	32.9 ± 4.9	28.5 ± 7.4	31.9 ± 5.9
Sex				
Male	10 (62.5)	5 (62.5)	10 (71.4)	4 (57.1)
Female	6 (37.5)	3 (37.5)	4 (28.6)	3 (42.9)
Ethnicity				
Hispanic or Latino	6 (37.5)	4 (50.0)	5 (35.7)	1 (14.3)
Not Hispanic or Latino	10 (62.5)	4 (50.0)	9 (64.3)	6 (85.7)
Race				
White	7 (43.8)	5 (62.5)	13 (92.9)	7 (100.0)
Black or African American	8 (50.0)	3 (37.5)	1 (7.1)	0 (0.0)
Asian	1 (6.3)	0 (0.0)	0 (0.0)	0 (0.0)
BMI (kg/m^2^)	30.27 ± 3.24	33.18 ± 4.18	24.21 ± 3.34	25.94 ± 3.68

Note

Values are presented as *n* (%) or mean ± *SD*.

Abbreviations: BMI, body mass index; *SD*, standard deviation; T1D, type 1 diabetes.

#### 3.1.2 Safety

Overall, there were 13 TEAEs reported in 8 patients (Table 2). Of these, 11 events in 6 patients (37.5%) were reported in the eptinezumab group and 2 events in 2 patients (25.0%) in the placebo group. Only one patient had a TEAE that was considered related to study treatment (eptinezumab group: mild abdominal pain [gas] and mild nausea). All TEAEs were mild in intensity, with the exception of one eptinezumab‐treated patient who experienced moderate headaches (not considered related to study drug) on three separate occasions. There were no TEAEs that led to treatment discontinuation and no serious TEAEs. There were also no clinically significant changes in vital signs, 12‐lead ECG parameters, physical examination results or clinical laboratory test results over time or between treatment groups. No patients reported suicidal ideation or behaviour during the study, and no patients developed antibodies to eptinezumab following treatment.

**TABLE 2:** Treatment‐emergent adverse events as *n* (%)

Adverse event	Study 1 (Overweight/Obese)		Study 2 (T1D)	
	Eptinezumab 100 mg *N* = 16	Placebo *N* = 8	Eptinezumab 100 mg *N* = 14	Placebo *N* = 7
Any TEAE	6 (37.5)	2 (25.0)	12 (85.7)	7 (100.0)
Serious TEAE	0	0	0	0
Study drug‐related TEAE	1 (6.3)	0	2 (14.3)	2 (28.6)
TEAE leading to study discontinuation	0	0	0	0
Most common events (≥2 patients in any treatment group)				
Hypoglycaemia	0	0	10 (71.4)	4 (57.1)
URTI	1 (6.3)	1 (12.5)	2 (14.3)	3 (42.9)
Abdominal pain	1 (6.3)	0	0	2 (28.6)
Diarrhoea	0	0	0	2 (28.6)

Abbreviations: T1D, type 1 diabetes; TEAE, treatment‐emergent adverse event; URTI, upper respiratory tract infection.

#### 3.1.3 Pharmacodynamic parameters

There was no significant difference in BMR (primary pharmacodynamic end‐point) between eptinezumab 100 mg and placebo at 7 days post‐administration (Table 3). The LS mean ± *SE* change in BMR from baseline to day 7 was 6.4 ± 32.9 Kcal/day in the eptinezumab group and ‒25.2 ± 47.3 Kcal/day in the placebo group. The LS mean difference (31.6 [95% CI ‒90.6, 153.8]) was not statistically significant.

**TABLE 3:** Basal metabolic rate change from baseline to day 7

Adverse event	Study 1 (Obese)	
	Eptinezumab 100 mg *N* = 16	Placebo *N* = 8
Basal metabolic rate (Kcal/day)		
Mean (*SE*)	1614.6 (42.7)	1749.8 (99.4)
Change from baseline to day 7		
LS mean (*SE*)	6.4 (32.9)	−25.2 (47.3)
Difference in LS means (*SE*)	31.6 (58.8)	
95% CI of LS mean difference	(−90.6, 153.8)	

Abbreviations: CI, confidence interval; LS, least squares; *SE*, standard error.

There was a modest difference observed in the total caloric intake between the eptinezumab and placebo groups at an ad libitum buffet meal following treatment (2156 Kcal vs 2022 Kcal, respectively). However, both groups reduced their total caloric intake from baseline (‒213.4 Kcal vs ‒315.7 Kcal, respectively) and the difference between groups (102.3 [(208.23] Kcal) was not statistically significant (95% CI ‒330.74, 535.35). There were no notable changes in any other secondary pharmacodynamic end‐points following treatment with eptinezumab (data not shown).

### 3.2 Patients with T1D (study 2)

#### 3.2.1 Patient demographics and disposition

A total of 21 patients (14 eptinezumab, 7 placebo) were enrolled into study 2; demographic characteristics are shown in Table 1. The mean age was 28.5 years in the eptinezumab group and 31.9 years in the placebo group, and the study population was predominantly male (71.4% and 57.1%, respectively). There were more patients of Hispanic ethnicity in the eptinezumab group (35.7%) compared with the placebo group (14.3%). One patient in the eptinezumab group withdrew for personal reasons prior to completion of the study (day 38), but was included in the safety and pharmacodynamic population. Overall, the mean duration of diabetes was 12.75 ± 5.9 years. Relevant concomitant medications included insulin (100%), statins (19%), angiotensin converting enzyme inhibitors (5%) and the anti‐obesity agent phenmetrazine hydrochloride (5%).

#### 3.2.2 Safety

There were 70 TEAEs reported in 19 patients (90.5%) in this study (Table 2). Of these, 34 events in 12 patients (85.7%) were reported in the eptinezumab group, and 36 events in 7 patients (100.0%) were reported in the placebo group. There were 10 separate treatment‐related TEAEs reported by 4 patients, including 2 patients (14.3%) in the eptinezumab group and 2 patients (28.6%) in the placebo group. For the two patients receiving eptinezumab, the treatment‐related TEAEs included hypoglycaemia, nausea, vomiting and upper respiratory tract infection (*n* = 1 each). With the exception of the upper respiratory tract infection event, which lasted 19 days, all treatment‐related TEAEs experienced with eptinezumab were transient (duration ≤1 day). For the two patients in the placebo group, treatment‐related TEAEs included hypoglycaemia (*n* = 2) and oral hypoesthesia (*n* = 1), both lasting <1 day. All of the TEAEs were mild or moderate in severity and there were no TEAEs that led to discontinuation. There were also no serious events or deaths reported in this study. The most frequently reported TEAE was hypoglycaemia, reported in 71% of patients in the eptinezumab group and 57% of patients in the placebo group. Only three patients experienced hypoglycaemia that was considered related to study medication (eptinezumab, *n* = 1; placebo, *n* = 2). There were also no changes in clinical laboratory test results, vital signs, 12‐lead ECG parameters or physical examination findings. Two patients were confirmed positive for the presence of anti‐eptinezumab antibodies, with both exhibiting low titre responses (≤213). Seroconversion occurred by either day 56 or day 84.

#### 3.2.3 Pharmacodynamic parameters

**FIGURE 1:**
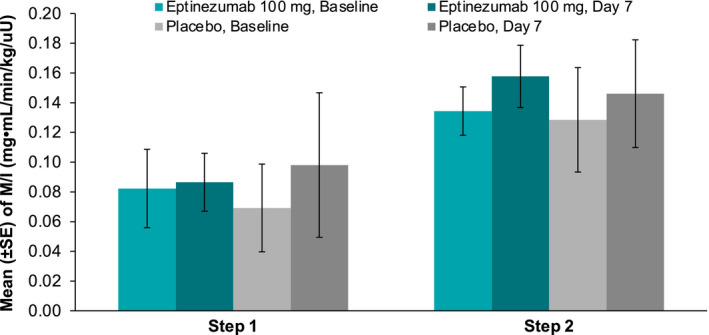
Mean M/I ratio at baseline and day 7 for eptinezumab and placebo. *Step 1*: Insulin was infused at a rate of 10 mU/m^2^/min for 180 min. This level of insulin infusion raised the plasma insulin concentration to a level suitable for assessing sub‐maximal suppression of endogenous glucose production (EGP). *Step 2*: Insulin was infused at a rate of 40 mU/m^2^/min for 180 min (beginning at the end of Step 1). This level of insulin infusion was sufficient to provide near‐maximal glucose disposal and EGP suppression. Abbreviations: M/I, weight‐corrected glucose infusion rate (GIR) divided by the plasma insulin concentration at steady state

**FIGURE 2:**
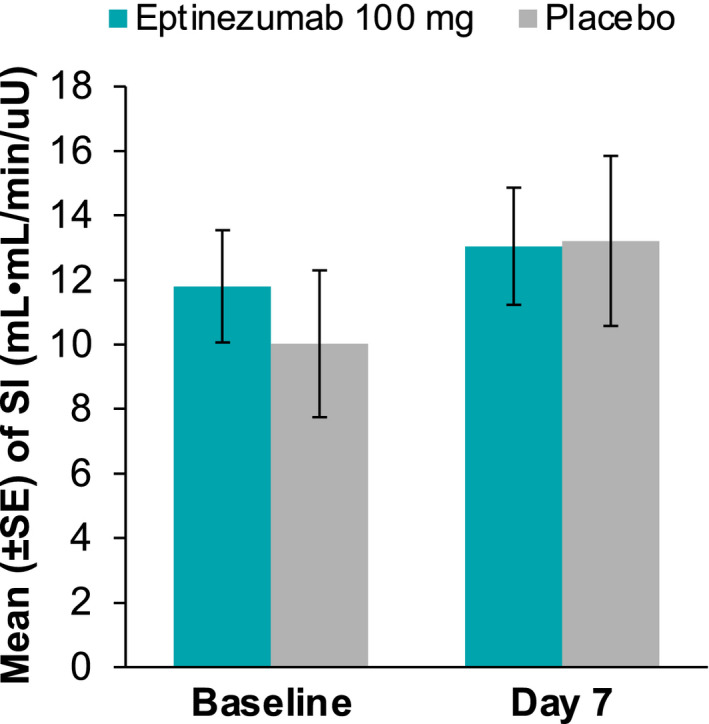
Mean sensitivity index value at baseline and day 7 for eptinezumab and placebo. Abbreviation: *SE*, standard error

**FIGURE 3:**
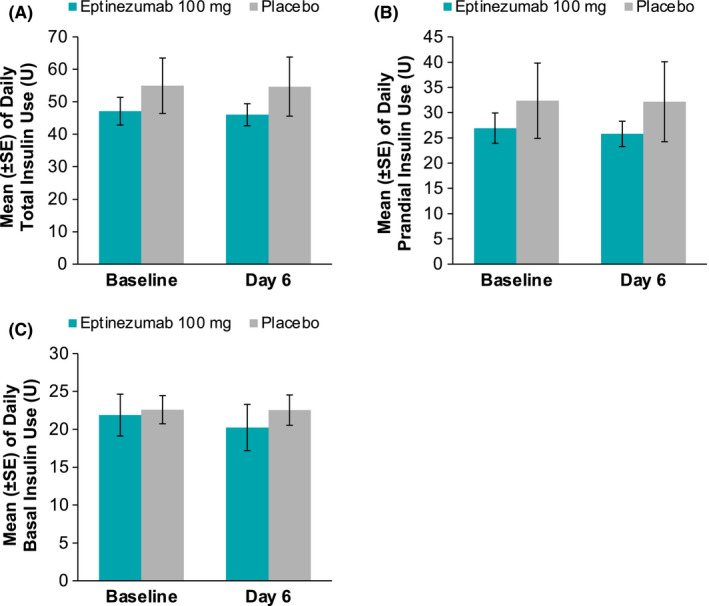
Mean daily insulin use at baseline and at day 6 for A, total insulin, B, prandial insulin, and C, basal insulin. Abbreviation: *SE*, standard error

There were no significant changes from baseline in the M/I ratios (primary pharmacodynamic parameter) for either the eptinezumab or placebo groups (Figure 1). During Step 1, the M/I ratio (LS mean ± *SE*) was 0.007 ± 0.025 (95% CI ‒0.045, 0.059) in the eptinezumab group and 0.024 ± 0.034 (95% CI ‒0.047, 0.095) in the placebo group. During Step 2, the M/I ratios were 0.024 ± 0.016 (95% CI ‒0.009, 0.056) and 0.017 ± 0.021 (95% CI ‒0.028, 0.062), respectively. There were also no significant between‐group differences for changes in M/I ratios (LS mean difference ± *SE*) from baseline to day 7 during Step 1 (‒0.017 ± 0.042; 95% CI ‒0.105, 0.071) or Step 2 (0.006 ± 0.026; 95% CI ‒0.049, 0.062). Similarly, there were no changes from baseline for other measures of insulin sensitivity (ie change from baseline to day 7 in the bodyweight‐corrected GIR or the insulin sensitivity index), and no differences between treatment groups for these parameters (Figure 2). There were also no changes from baseline in the total, prandial or basal insulin requirements in the eptinezumab or placebo groups (Figure 3A–C) and no differences between groups.

At baseline, patients in the eptinezumab group had a numerically higher percentage of time in the hyperglycaemic range (72.6% vs 65.2%), and lower percentages of times in the euglycemic (26.5% vs 33.2%) and hypoglycaemic (0.9% vs 1.7%) ranges, compared with the placebo group. At day 6, eptinezumab‐treated patients had a tendency to spend more time in the euglycemic (39.7%) and hypoglycaemic (2.5%) ranges and less time in the hyperglycaemic range (57.8%), whereas placebo‐treated patients showed minimal change (62.1% hyperglycaemic, 35.5% euglycemic, 2.3% hypoglycaemic). This suggests a nominal trend towards less time in hyperglycaemia following treatment with eptinezumab, though the differences between arms are not large enough to rule out this being a random event.

## 4 DISCUSSION

Treatment of migraine in patients who are overweight/obese or who have T1D can be challenging. Many conventional migraine prevention agents, such as propranolol, divalproex sodium and amitriptyline, have been shown to cause weight gain in patients with migraine.^22^, ^23^ Although, to date, there are no published data evaluating the impact of CGRP inhibitor mAbs in overweight/obese patients, nor in patients diagnosed with T1D, it is important to assess drug safety and physiologic effects in these populations due to the role played by CGRP in glucose metabolism.

In the current studies, eptinezumab was well tolerated in otherwise healthy overweight/obese patients and in those with T1D. Incidence rates of TEAEs were generally similar between eptinezumab‐treated patients and those receiving placebo, and the majority of events were mild in severity. Although there was a higher incidence of TEAEs in the T1D study, many of the reported events were related to the underlying disease. For example, 38/70 events in the T1D study were hypoglycaemia events, and, of these, several were reported during the follow‐up period when patients were not in the clinical unit. Hypoglycaemia is a common AE in patients with T1D, generally due to an imbalance between caloric supply and circulating insulin levels^24^; thus, hypoglycaemic events were not unexpected in this population. There were no serious TEAEs or events that resulted in treatment discontinuation in either study, and no new safety signals were identified. Immunogenicity responses were not observed in overweight/obese patients and at a low rate (14%) in patients with T1D. These results are consistent with the findings of the phase 3 trials in episodic migraine (*n* = 888)^20^ and chronic migraine (*n* = 1072)^21^ populations, in which eptinezumab was found to be well tolerated, with most TEAEs being mild or moderate in severity.

In preclinical studies, an analogue of eptinezumab (ALD405) was found to improve whole‐body insulin sensitivity, hepatic insulin sensitivity and skeletal muscle insulin sensitivity in rats [data on file]. The metabolic improvements were observed both in rats that were normo‐insulinemic/normoglycemic and that had normal insulin sensitivity, and in rats with insulin resistance that were hyperinsulinemic (but not hyperglycaemic). Other published preclinical studies have shown that inhibition of CGRP using antibodies increases insulin secretion in mice, and extends first‐phase insulin secretion.^25^

In the current clinical analysis in overweight/obese patients, eptinezumab had no significant effect on BMR or other measures of energy metabolism, and there was also no effect versus placebo on food intake. This may have important clinical relevance, because treatment that avoids weight gain is advantageous for patients, and this attribute may help to increase treatment compliance within the migraine population. Furthermore, in patients with T1D, eptinezumab had no significant effect on glucose metabolism (according to the measured M/I ratios). These data suggest that eptinezumab can be administered to these populations without concern for adverse metabolic consequences. In addition, the IV route of administration may provide additional benefit for treating overweight and obese patients. Eptinezumab, delivered IV, demonstrates rapid onset and achieves 100% bioavailability immediately at the end of infusion.^19^ Evidence indicates that the slow absorption rate following subcutaneous administration may not be as effective when rapid onset of action is required.^26^, ^27^ Drawbacks of subcutaneous administration include the incomplete bioavailability after subcutaneous administration.^26^, ^28^ Following IV administration, a biotherapeutic is directly injected into the systemic circulation. Following subcutaneous administration, however, the biotherapeutic is injected into the extracellular space of the subcutaneous tissue; from there it has to be transported to blood or lymph capillaries for absorption, prior to reaching systemic circulation.^26^

The limitations of these studies are the small sample sizes involved. Moreover, these studies included a predominance of males in the analysis populations, which is in contrast to the higher prevalence of migraine among females within general clinical practice.^2^, ^29^

## 5 CONCLUSION

The data from these studies suggest that eptinezumab is well tolerated in healthy patients who were overweight/obese and those with T1D. Further, eptinezumab had no significant effect on the change in BMR, and overall metabolic parameters, in overweight/obese patients, and had no significant effect on insulin sensitivity in patients with T1D. Overall, these results provide support for the use of eptinezumab in these important subgroups of patients with migraine.

## CONFLICT OF INTEREST

B. Baker and S. Pederson are full‐time employees of Lundbeck Seattle BioPharmaceuticals. B. Schaeffler was a full‐time employee of Lundbeck Seattle BioPharmaceuticals at the time of study and during manuscript preparation. J. Hirman is a contracted service provider of biostatistical resources for Lundbeck Seattle BioPharmaceuticals. M. Hompesch was a contracted service provider of Alder BioPharmaceuticals (now known as Lundbeck Seattle BioPharmaceuticals) at the time of study and is the Editor‐in‐Chief for *Endocrinology, Diabetes & Metabolism*. J. Smith was a full‐time employee and stockholder of Alder BioPharmaceuticals (now known as Lundbeck Seattle BioPharmaceuticals) at the time of study and was a contracted service provider for Lundbeck Seattle BioPharmaceuticals during manuscript development.

## AUTHOR CONTRIBUTIONS

B. Baker, B. Schaeffler, S. Pederson and J. Smith contributed to the conception and/or design of the study. J. Hirman contributed to data analysis. All authors contributed to data interpretation, reviewed and provided critical revision of all manuscript drafts for important intellectual content, and read and approved the final manuscript for submission.

## Data Availability

The data reported are part of an ongoing, global sponsor‐led clinical development and registration programme. Deidentified participant data are not available for legal and ethical reasons.
